# TiO_2_ Containing Hybrid Composite Polymer Membranes for Vanadium Redox Flow Batteries

**DOI:** 10.3390/polym14081617

**Published:** 2022-04-15

**Authors:** Gowthami Palanisamy, Tae Hwan Oh

**Affiliations:** School of Chemical Engineering, Yeungnam University, Gyeongsan 38541, Korea

**Keywords:** vanadium redox flow battery, hybrid membrane, cation exchange membrane, TiO_2_, inorganic additive, physicochemical properties, ion selectivity

## Abstract

In recent years, vanadium redox flow batteries (VRFB) have captured immense attraction in electrochemical energy storage systems due to their long cycle life, flexibility, high-energy efficiency, time, and reliability. In VRFB, polymer membranes play a significant role in transporting protons for current transmission and act as barriers between positive and negative electrodes/electrolytes. Commercial polymer membranes (such as Nafion) are the widely used IEM in VRFBs due to their outstanding chemical stability and proton conductivity. However, the membrane cost and increased vanadium ions permeability limit its commercial application. Therefore, various modified perfluorinated and non-perfluorinated membranes have been developed. This comprehensive review primarily focuses on recent developments of hybrid polymer composite membranes with inorganic TiO_2_ nanofillers for VRFB applications. Hence, various fabrications are performed in the membrane with TiO_2_ to alter their physicochemical properties for attaining perfect IEM. Additionally, embedding the -SO_3_H groups by sulfonation on the nanofiller surface enhances membrane proton conductivity and mechanical strength. Incorporating TiO_2_ and modified TiO_2_ (sTiO_2_, and organic silica modified TiO_2_) into Nafion and other non-perfluorinated membranes (sPEEK and sPI) has effectively influenced the polymer membrane properties for better VRFB performances. This review provides an overall spotlight on the impact of TiO_2_-based nanofillers in polymer matrix for VRFB applications.

## 1. Introduction

Fossil fuel energies have been commonly used as an energy source for stationary, mobile, and transport applications for the past several decades [1,2,3,4]. However, fossil fuel energy is associated with numerous disadvantages such as air pollution and greenhouse gas emissions [5,6,7]. Apart from the environmental concern, fossil fuel depletion has a significant impact on industrialization and globalization [8]. Fossil fuels are highly extracted from the earth because of their higher necessity for the present energy needs. The worldwide population growth is rapidly increasing, which is also a significant reason for the higher extraction and utilization of fossil fuels. The significant population growth created energy shortage-related issues [9,10]. Moreover, the emission of greenhouse gases such as CO_2_, CH_4_, SO_2,_ and N_2_O are the major byproducts of fossil fuels during the energy conversion process [5,11,12]. The byproducts of fossil fuels create serious environmental pollution and increase the Earth’s atmospheric temperature [13,14,15]. To overcome fossil fuel-related issues such as fossil fuel depletion, energy shortage, and greenhouse gas emissions, worldwide researchers are searching for various kinds of renewable and alternative energy resources. The development of alternative energy needs to be green and clean for the environment. The excellent energy sources of solar, wind, hydro, geo, and bio energy have been effectively considered renewable and alternative energies for the present and future [16,17,18,19,20]. The energy derived from the primary renewable energy resources can be stored and utilized using various energy conversion and storage devices such as electrochemical systems (fuel cells, supercapacitors, and batteries) [21,22,23]. In fuel cells, the electricity is generated from hydrogen or other fuels (chemical energy) in a cleaner process by a pair of redox reactions [4,24]. The energy can be stored electrostatically (Helmholtz layer) and electrochemically (Faradic charge transfer) in supercapacitors [25,26]. Secondary batteries are considered one of the most efficient energy systems among the different energy conversion and storage systems because of their reliable charge and discharge process [27,28]. In batteries, chemical energy is converted into electrical energy by one or more electrical cells, where the redox reaction occurs between the two electrodes. The most important advantage of secondary batteries is that they can be effectively recharged many times and are reusable. The secondary batteries can be classified into many categories such as solid batteries, liquid batteries, and gel batteries [29,30,31,32,33,34]. The secondary batteries are widely used in energy devices and portable electronics such as uninterrupted power supplies, hybrid electric vehicles, mobiles, laptops, etc. Among the different categories of batteries, the redox flow batteries show considerable advantages on an industrial scale [35,36]. The energy storage in a redox flow battery is entirely different from the enclosed batteries such as lithium-ion batteries and lead-acid batteries. In enclosed batteries, the energy is stored in electrode materials. The energy is stored in the electrolyte solution (soluble and fluid electroactive species) in a redox flow battery [37,38]. Commonly, the polymer-based ion exchange membrane plays a significant role in redox flow batteries (RFB) such as controlling the cross-over of vanadium electrolytes between the anode and cathode and facilitating the proton transportation during the charge and discharge process (catholyte to anolyte or anolyte to catholyte) [39,40,41].

This review focus on the advantages and developments of hybrid membranes for vanadium redox flow battery (VRFB) applications. A systematic overview representing the importance of VRFB, VRFB components, and the primary classification of membranes is provided. In this comprehensive, we reviewed types of membrane candidates for VRFB and recent examples of TiO_2_ nanofiller in the different kinds of polymers, namely Nafion, sulfonated polyimide (sPI), and sulfonated poly(ether ether ketone) (sPEEK). To understand the impact of TiO_2_ in the membrane matrix, we explained the performances of hybrid membranes based on the unit cell results from the relevant studies.

## 2. Importance and Components of Vanadium Redox Flow Batteries

In the battery sector, the RFBs have been considered an efficient energy system because of their possible advantages such as eco-friendly system, high energy storage system, modification of required size/design of electrolyte tank, easier to scale-up process, room temperature operation, considerable cycle performances, and efficient energy efficiency [41,42,43,44]. The presence of electroactive species in the electrolyte solution is used to store or release the energy during the VRFB unit cell operation. According to the electrolyte solution (solvents and/or electroactive species) in the anolyte and catholyte, and the design of the redox flow battery, it can be classified into various categories. Based on the electroactive species solvents, it can be classified as a non-aqueous redox flow battery, an aqueous redox flow battery, and a hybrid aqueous/non-aqueous redox flow battery [45,46,47,48]. More interestingly, the RFB has been further classified based on their electroactive species (redox couple) presence in the electrolyte solution such as Hydrogen/Bromine [49], Iron/Chromium [50], Vanadium/Bromine [51], Zinc/Bromine [52], All-Vanadium [53,54], Vanadium/Polyhalide [55], Zinc/Cerium [56], Iron/Vanadium [57], Tin/Bromine [58], Polysulfide/Bromine [59], vanadium/Manganese [60], Cerium/Lead [61], Soluble Pb [62], Zinc/Polyiodide [63], aqueous organic [64,65], and non-aqueous organic RFBs [66,67].

**Figure 1:**
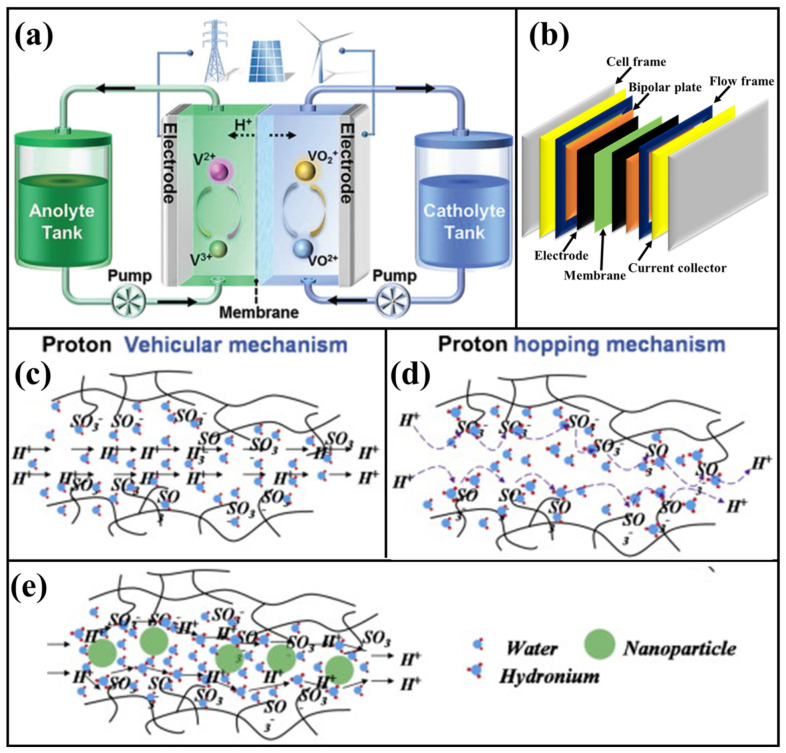
(**a**) Schematic representation of VRFB unit cell design and energy conversion process. Reprinted with permission from ref. [68]. Copyright © 2020 WILEY-VCH Verlag GmbH & Co. KGaA, Weinheim. (License Number: 5270131060091). (**b**) Schematic illustration of VRFB unit cell components. Proton transport mechanisms: (**c**) vehicular mechanism and (**d**) hopping mechanism. (**e**) Proton transport possibilities in polymer-nanoparticle hybrid composite membrane. Reprinted with permission from ref. [94]. Copyright © 2014 The Korean Society of Industrial and Engineering Chemistry. Published by Elsevier B.V. (License Number: 5270141297621).

Compared to different RFB systems, the VRFB is considered an efficient battery system because of the different oxidation states of vanadium electroactive species (vanadium ions: V^2+^, V^3+^, VO^2+^, and VO_2_^+^) used in the electrolyte solution in anolyte and catholyte tanks as shown in Figure 1a [48,68,69,70]. Moreover, it possesses numerous benefits such as an efficient option for large-scale energy storage, reasonable discharge capacity, cycle life, high safety, room temperature operation, and environmental friendliness [70,71,72]. Thus, the VRFB has already proved its performance in the large-scale energy system (0.3 MW/1.3 MWh, 0.45 MW/1.44 MWh, and 2 MW/8 MWh) [69,73]. To improve the VRFB’s overall performance, intense research focuses on the major components, namely electrodes, bipolar plates, membranes, and electrolytes. The performance of VRFB can be altered by employing the co-solvents and different kinds of additives in the electrolyte solution for boosting the steadiness, electroactive species solubility, and overall electrochemical performances. Different kinds of additives have been utilized as stabilizing agents such as zwitterion-type molecule (pyridinium propyl sulphobetaine with sulfonic and pyridine groups), inositol or phytic acid, Trishydroxymethyl aminomethane, Polyacrylic acid, l-glutamic acid, antimony ions, sodium formate, methanesulfonic acid, K_2_SO_4_, Li_2_SO_4_, KHSO_4_, Na_2_SO_4_, CH_3_SO_3_H, MgCl_2_ for different oxidation states of vanadium ions [74,75,76,77,78,79,80,81]. In VRFB, the bipolar plate serves as a multifunctional component for constructing and operating the unit cell system [82,83,84]. The major role of the bipolar plate is collecting the current (electron pathway), distributing electrolytes to the electrodes via flow field, internally connecting every single cell in the stacked VRFB unit cell, acting as structural support to the stack, and chemically discrete every single cell. The choice of bipolar plate materials is most important because of their working condition in high acidic conditions (~3M H_2_SO_4_) and various applied potentials [82,83]. The developments of bipolar plates for VRFB are mainly classified into three categories, namely graphitic, metallic, and carbon polymer composites bipolar plates [82,83,84,85,86,87]. The following essential component in VRFB is electrode materials, which is the component of direct contact with electrolyte that induces the vanadium redox reaction that occurs during the charge and discharge process [88,89,90,91]. The electrode materials possess the properties of excellent chemical stability, higher electrochemical active sites, and greater electrical conductivity behavior, which can effectively serve as efficient electrode materials for the VRFB system. Carbon felt, carbon nanofiber, graphite felt, and carbon paper have been commonly studied as efficient electrode materials for VRFB. To further improve the stability and electrochemical reactions, the electrodes were modified with different concepts such as heteroatom doped, metal oxide nanoparticle decoration, graphene deposition, functionalization with SO_3_H, and halogen atom doped [88,89,92,93]. The center part of the VRFB is a polymer membrane, which is one of the most significant components of the device. The primary function of polymer membrane in VRFB is to separate the anode and cathode part, transport the proton from anode to cathode or cathode to anode side during the unit cell operation, and control the crossover of vanadium ion.

## 3. Types of Membranes for Vanadium Redox Flow Batteries

For VRFB application, different categories of polymer membrane have been developed based on the polymer source material, functional group, chemical structure, design, and composition. The membrane materials have been derived from different sources and named natural, semi-synthetic, and synthetic polymers [95]. According to the polymer functional properties, the VRFB membranes can be classified as non-ionic porous membranes, anion exchange membranes, cation exchange membranes (CEM), mosaic ion exchange membranes, bipolar ion-exchange membranes, and amphoteric ion-exchange membranes [69]. Additionally, the membrane has also been classified based on the group of polymers mainly fluoro-carbon, hydro-carbon, and N-heterocyles [96,97]. There are different kinds of commercial membranes that exist through different suppliers for VRFB systems such as DuPont (Wilmington, DE, USA) (Nafion NR211, NR212, N112, N115, N117, and N1135), Solvay (Belgium, Brussels) (Aquivion E98–05 and Aquivion E87–05S), Tokuyama Corp. (Tokyo, Japan) (Neosepta CM-1), W.L Gore & Associates (Newark, DE, USA) (Gore Select L-01854 and Gore Select M-04494), Fumatech (Bietigheim-Bissingen, Germany) (Fumasep F-1850, Fumasep FAP-PP-475, and Fumasep FAP-420), and ASAHI Glass (Tokyo, Japan) (Selemion HSV, Selemion HSF, Selemion CMV, Selemion AMV, and APS) [35,96,97]. Apart from the numerous advantages, most commercial membranes possess high production costs, which significantly affects the benefits of VRFB in terms of commercial perspective. To overcome the cost-related issues, researchers worldwide are developing different concepts to decrease the cost of high-performance commercial polymers and study the different cost-effective polymers. The developments include modification of commercial membranes [98,99,100], blend membrane [101,102,103] composite membrane [104,105,106], coated membrane [107,108], core–shell membrane [109,110], ionomer reinforced membrane [111,112], grafted polymer membrane [36,112,113], cross-linked membrane [114,115,116], and acid-base polymer membrane [117,118].

Mostly, the polymer-based cation-exchange membranes transfer the protons through two mechanisms (Grotthus mechanism—hopping, and Vehicular mechanism—diffusion) [94,97,119,120]. The proton transport in polymer membrane mainly occurs through counter ion (sulfonation (–SO_3_H) functional group), ionic cluster formation (proton channel), and water molecules as shown in Figure 1c–e. Apart from the several advantages (such as high proton transportation), most polymer membranes are reasoned for higher vanadium ion crossover, which significantly affects the overall VRFB performances. To overcome this barrier, attempts were made with different concepts. The development of a new polymer membrane with inorganic polymer and additives shows considerable attention to decreasing the membrane cost, controlling the vanadium ion permeability, improving selectivity, and enhancing stability. Different dimensions of inorganic nanomaterials, functionalized inorganic fillers and inorganic polymers such as silica (SiO_2_) [121,122], sulfonated SiO_2_ [105], Titanium oxide (TiO_2_) [123,124], sulfonated TiO_2_ [125,126], molybdenum disulfide (MoS_2_) [127], tungsten oxide (WO_3_) [128], graphene oxide (sulfonated and amine-functionalized) [129], sulfonated multi-wall carbon nanotubes (s-MWCNTs) [130], cerium zirconium oxide nanotube (Ce_2_Zr_2_O_7_) [131], sulfated zirconia [132], functionalized silicon carbide (SiC) [133], boehmite (AlOOH) [134], oxidized g-C_3_N_4_ [135], metal-organic framework (MOF-UiO-66) [136], N-(trimethoxysilylpropyl)-N,N,N-trimethylammonium chloride [137], and alkoxy silane functionalized polymer [104] have been effectively considered as an additive to develop the organic–inorganic hybrid membranes. In the recent period, there has been a growing interest in TiO_2_ as a filler in different polymer membranes (Nafion [68,123,138], sulfonated polyimide (sPI) [139], and sulfonated poly(ether ether ketone) (sPEEK) [124,125,126,140,141,142,143] for VRFB system. The inorganic filler of TiO_2_ has been considered one of the efficient additives to VRFB membranes because of its advantages such as low cost, availability, stable metal oxide, high chemical stability, antioxidant ability, and hydrophilicity (rich hydroxyl groups) [68,125,126,139]. Moreover, the TiO_2_ properties have been further altered through modification of functional properties, and surfaces such as sulfonated and organic silica modified TiO_2_, which further influences the compatibility, and excellent dispersion of TiO_2_ in the membrane matrix [125].

## 4. Nafion-TiO_2_ Hybrid Polymer Membrane for Vanadium Redox Flow Batteries

**Figure 2:**
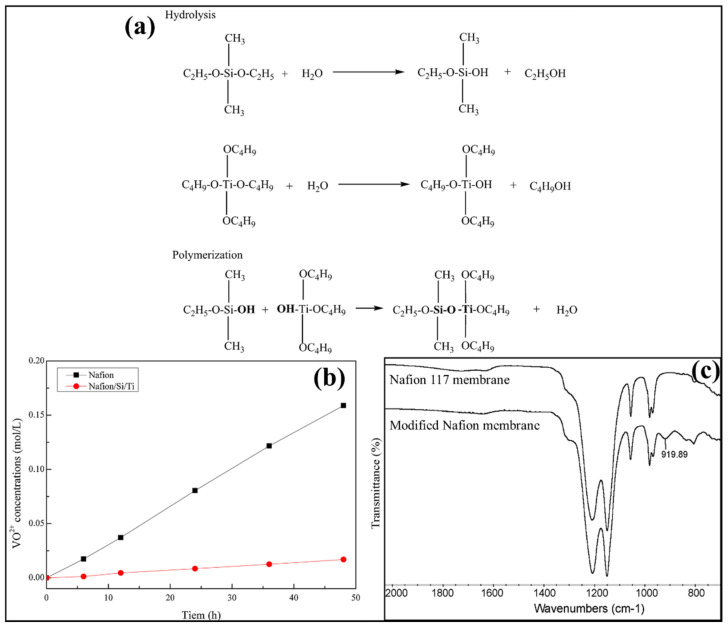
(**a**) Schematic representation of organic silica modified TiO_2_. (**b**) Vanadium ion crossover and (**c**) FTIR analysis of Nafion and modified Nafion membranes. Reprinted with permission from ref. [138]. Copyright © 2009 Elsevier B.V. (License Number: 5270100780394).

The perfluorosulfonic acid ionomer with a long-side chain (Nafion membrane) is commonly used as one of the efficient membrane candidates for the VRFB application because of its higher proton conductivity and excellent chemical stability [144,145]. However, the production cost and vanadium ion crossover due to larger-sized ion cluster formation limits its advantages [146,147,148]. To overcome the higher vanadium ion crossover-related issues, different kinds of inorganic fillers were introduced in the Nafion polymer through various concepts for developing the hybrid membrane. In this connection, efficient attempts have been made with TiO_2_ introduction in Nafion polymer to control the vanadium permeability in Nafion membranes. Teng et al. developed Nafion/Organic silica modified TiO_2_ composite membrane by sol–gel method and used it for VRFB applications to overcome the solubility issue of TiO_2_ in the Nafion/TiO_2_ hybrid membrane [138]. Figure 2a illustrates the hydrolysis and polymerization reactions formed during the Nafion/organic silica modified TiO_2_ composite membrane preparation. Due to the filling of modifiers (Organic silica modified TiO_2_ nanoparticles) into Nafion membrane polar clusters, the modified Nafion membrane exhibited increased thickness with the decrease in water uptake and IEC than the Nafion membrane. Organically modified TiO_2_ into the Nafion clusters in the composite membrane exhibited lower vanadium permeability than the Nafion membrane as shown in Figure 2b. It has been observed that approximately 1.7 mL of water was transferred in the modified Nafion membrane while 2.7 mL of water was transferred across the unmodified Nafion membrane. As a result, the modified Nafion membrane exhibited greater water transport resistance than the unmodified Nafion membrane, which was reasoned by the presence of inorganic additives in the membrane. The Si and Ti distribution and titania–silica phase formation has been observed inside the modified Nafion membrane. Interestingly, a new Si-O-Ti vibration peak (in Figure 2c) was observed at 919.89 cm^−1^ in the modified Nafion membrane, which confirms the composite membrane. As shown in Figure 2b, VRFB with the modified Nafion membrane exhibited lower vanadium crossover, which can exhibit higher discharge capacity than the Nafion membrane. Concurrently, the larger area resistance of the modified Nafion membrane increased IR drop resulting in a little higher average charge voltage. The coulombic efficiency of 94.8% and 90.8% was seen in VRFB with a modified Nafion membrane and Nafion membrane, respectively. As a result of increased area resistance in the modified composite membrane, it exhibited little lower voltage efficiency of 82.2% than the Nafion membrane, 84.8%. Therefore, in the VRFB application, an appropriate amount of Si/Ti content can be used for modified Nafion membrane preparation. The self-discharge of the VRFB single cell through open-circuit voltage (OCV) after being charged to SOC at 75% of the modified Nafion membrane showed better performance than the Nafion membrane. The modified Nafion membrane maintained OCV above 65 h beyond 0.8V, higher than the unmodified Nafion membrane (30 h). This resulted from the modified Nafion membrane exhibiting lower vanadium ions crossover between the compartments in the VRFB system. Furthermore, the VRFB single cell with modified Nafion membrane up to 100 cycles exhibited no CE decay but a slight EE reduction. This shows the high stability of the modified Nafion membrane in highly acidic vanadium electrolyte solution [138].

Nafion/TiO_2_ hybrid membrane gained attention for VRFB applications in order to reduce vanadium ions permeation. Thus, Wang et al. prepared Nafion/TiO_2_ hybrid membranes by a practical and simple hydrothermal method and investigated their performances in VRFB cells [123]. The introduction of TiO_2_ by hydrothermal synthesis into polar clusters does not intrude the ion exchange groups (-SO_3_H) of the Nafion membrane. Thus, the Nafion/TiO_2_ hybrid membrane exhibited nearly the same IEC value of 0.85 mmol g^−1^ as the Nafion membrane. Due to partial filling of Nafion pores by TiO_2_ particles, Nafion/TiO_2_ hybrid membrane showed lower water uptake of 19.13% than the Nafion membrane. In the presence of an 8.3% TiO_2_ modifier, there has been a slightly increased area resistance in Nafion/TiO_2_ hybrid membrane. After membrane modification, the hybrid membrane exhibited lower vanadium permeability of 6.72 × 10^−6^ cm^2^ min^−1^ than the unmodified Nafion membrane (2.26 × 10^−5^ cm^2^ min^−1^) due to the partial filling of TiO_2_ particles in the Nafion membrane polar clusters. The Nafion/TiO_2_ hybrid membrane exhibited excellent chemical stability confirmed by XRD patterns. As per the degree of self-discharge of the VRFB unit cell, OCV for both the membranes gradually decreases at first and then sharply drops. The time for OCV of VRFB with hybrid membrane was nearly 37 h beyond 1.2 V, which was higher than Nafion membrane (14 h). This was mainly attributed to the lower vanadium crossover of the Nafion/TiO_2_ hybrid membrane. As a result, VRFB unit cells with the hybrid membrane exhibited higher CE (88.8%) and EE (71.5%) than the Nafion membrane [123].

**Figure 3:**
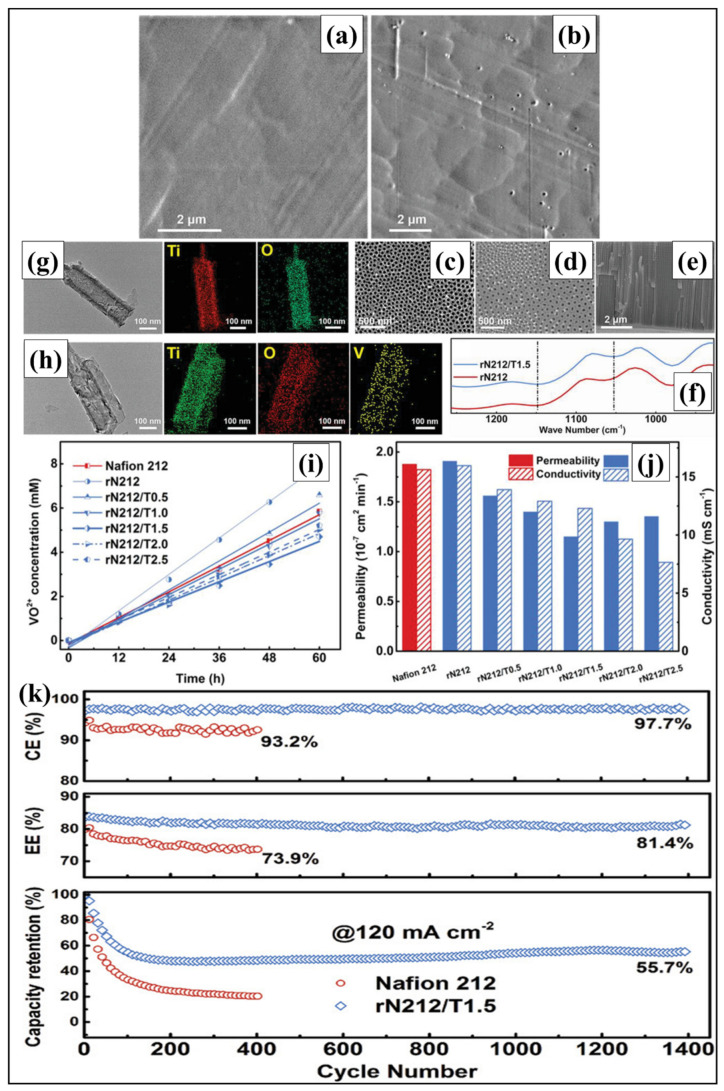
SEM cross-sectional view of (**a**) recast Nafion and (**b**) hybrid (rN212/T1.5) membranes. TiO_2_ nanotube arrays: (**c**) top, (**d**) bottom, and (**e**) cross-sectional view of FE-SEM. (**f**) FTIR results of recast Nafion and hybrid (rN212/T1.5) membrane. (**g**,**h**) TEM and EDS mapping of TiO_2_ nanotube—before and after soaking in VRFB electrolyte. (**i**) V^4+^ ion cross-over, (**j**) vanadium permeability, and proton conductivity of membranes. (**k**) VRFB cell test of recast Nafion and rN212/T1.5 membranes. Reprinted with permission from ref. [68]. Copyright © 2020 WILEY-VCH Verlag GmbH & Co. KGaA, Weinheim. (License Number: 5270131060091).

In another approach, Ye et al. (2020) developed a hybrid membrane by solution casting method with superhydrophilic TiO_2_ nanotubes dispersed in the matrix of Nafion for VRFB applications [68]. The surface and cross-sectional images of the recast Nafion (rN212) and hybrid membrane (rN212/T1.5) were examined through SEM (Figure 3a–h) for the confirmation of nanotube distribution in the hybrid membrane. Here, many pinholes with a heavy and compact structure are observed in rN212, while the size and amount were lowered in the rN212/T1.5 membrane due to TiO_2_ blending. Meanwhile, TiO_2_ blending creates straight rods with regular round holes in the rN212/T1.5 membrane. Predominantly, the shorter TiO_2_ nanotubes from long tubes during stirring and ultrasonication are responsible for rN212/T1.5 membrane mechanical strength. The chemical binding and dispersion of Ti^4+^ ions in the Nafion matrix improve the mechanical and chemical stability of the rN212/T1.5 membrane. The water uptake and swelling properties of the rN212/T1.5 membrane are lower than Nafion 212 membrane due to TiO_2_ nanotubes filling the Nafion pinhole matrix, which lowered the water absorption and polymer swelling. The reduced vanadium ion behavior of the rN212/T1.5 membrane was attributed to the impassable and stretchable ion pathway during TiO_2_ nanotube blending. Meanwhile, the high loading of nanotubes increases vanadium permeation by nanoparticle aggregation. Additionally, the rN212/T1.5 membrane observed decreased conductivity due to the parallel alignment of the nanotube on the membrane surface, which possibly blocked the proton transport. Concurrently, the rN212/T1.5 membrane has higher ion selectivity of 10.68 × 10^4^ S min cm^−3^ than the Nafion 212 membrane 8.32 × 10^4^ S min cm^−3^ as shown in Figure 3j. Hence, it has been suitable for VRFB applications. The mechanical strength of the rN212/T1.5 membrane (26.54 MPa) is comparable to the Nafion 212 membrane (27.31 MPa). It has been observed that the OCV of VRFB with rN212/T1.5 membrane sustained a longer time than the Nafion membrane. This is because of lower vanadium crossover by nanotube incorporation. It has been identified that the rN212/T1.5 membrane shows CE of 98.3% and EE of 84.4% higher than Nafion 212 membrane, which has stable performances after 1400 cycles as shown in Figure 3k. Thus, the rN212/T1.5 membrane exhibited long-term stability during VRFB operation [68].

## 5. Polyimide Polymer with TiO_2_ for Vanadium Redox Flow Batteries

Among different polymer-based non-fluorinated membranes, sPI membranes gained promising attraction in VRFB applications owing to their outstanding chemical stability, high selectivity, low vanadium permeation, and low cost with mechanical and thermal stability [139,149,150,151]. Despite this, sPI exhibited lower antioxidant and proton conductivities in VRFB application. To overcome this issue, Li et al. incorporated mesoporous TiO_2_ filler material into the sPI membrane for enhancing antioxidant ability and membrane proton transport by the blending method [139]. The successful blending of TiO_2_ into the sPI was indicated by bright points (size < 1.0 µm) on the surface of the sPI/TiO_2_ membrane and the amorphous nature of the composite membrane was obtained. Although the presence of an inorganic TiO_2_ component in the composite membrane enhances its chemical stability, the composite membrane exhibited lower water uptake (32.94%) and swelling ratio than the pure sPI membrane. The reason behind this involves the presence of minimal hydrophilic nature of TiO_2_ reduces water absorption and swelling. In proton conductivity, the composite sPI/TiO_2_ membrane (3.12 × 10^−2^ S cm^−1^) showed higher than the pure sPI membrane (2.47 × 10^−2^ S cm^−1^). This was due to the collegial effect of the mesoporous hydrated inorganic TiO_2_ particle and hydrated sulfonic group promoting the transport of protons in the composite membrane through adsorbing water by mesoporous TiO_2_. An increase in the degree of sulfonation of sPI enhances the proton conductivity value, but the swelling affects the membrane stability. Due to the improved size strength of the composite membrane, the vanadium ions permeability of 2.02 × 10^−7^ cm^2^ min^−1^ was observed, which was lesser than the Nafion 117 membrane (17.10 × 10^−7^ cm^2^ min^−1^). Additionally, the sPI/ TiO_2_ composite membrane (1.54 × 10^−5^ S min cm^−3^) exhibited more ion selectivity than the pure sPI (1.30 × 10^−5^ S min cm^−3^) and the Nafion 117 (0.34 × 105 S min cm^−3^). The sPI/TiO_2_ composite membrane performance in VRFB was performed with different current densities from 20 to 80 mA cm^−2^. As a result, the sPI/TiO_2_ composite membrane exhibited higher charge and discharge capacities than the Nafion 117 membrane due to the reduced vanadium ion permeability of the composite membrane. VRFB with an sPI/TiO_2_ membrane at the same current density showed higher CE than the Nafion 117 membrane. Furthermore, the sPI/TiO_2_ membrane and the Nafion 117 membrane exhibited adjacent VE at various current densities. At low current density, the sPI/TiO_2_ membrane displayed higher EE than the Nafion 117. For the sPI/TiO_2_ membrane, about 160 h, the OCV decreases up to 1.3 V and thus 65 h only for the Nafion 117 membrane. Meanwhile, CE, VE, and EE remain unchanged for up to 50 cycles of VRFB with the sPI/TiO_2_ membrane. This indicated the high durability, and chemical and cyclic stability of the sPI/TiO_2_ membrane, achieved by the incorporation of TiO_2_ [139].

## 6. Sulfonated Poly(Ether Ether Ketone)—TiO_2_ Based Hybrid Composites for Vanadium Redox Flow Batteries

As an alternative to a commercial membrane for VRFB, the sPEEK membrane obtained attention due to its chemical, mechanical and thermal stability with low cost, low vanadium ion permeability, and easy preparation. Increasing the degree of sulfonation in sPEEK creates a broad water channel in sPEEK that increases the membrane swelling, resulting in increased vanadium permeability and decreased membrane mechanical stability. Here, inorganic particles are introduced into the sPEEK membrane to reduce water channel size and increase the mechanical stability of the membrane.

**Figure 4:**
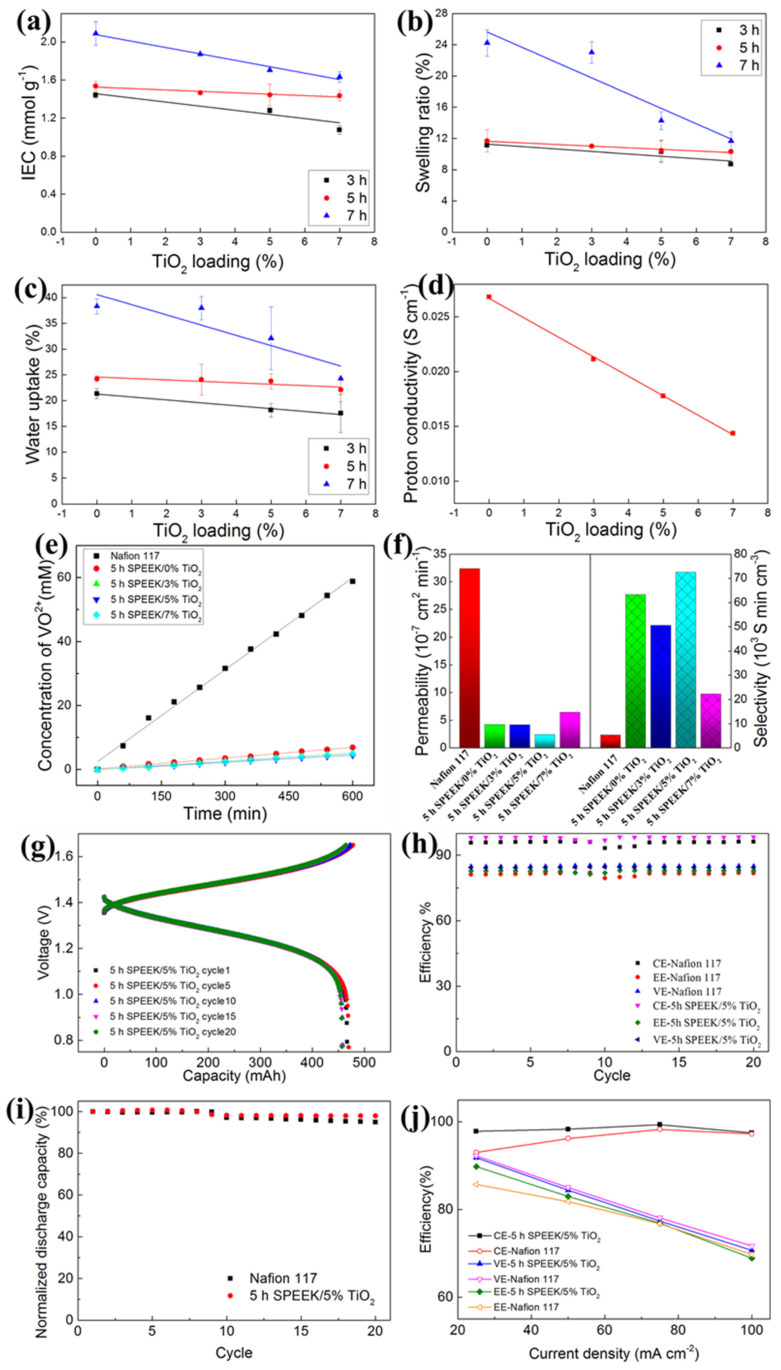
Different DS and amount of TiO_2_ nanoparticles in sPEEK/TiO_2_ composite membranes: (**a**) ion exchange capacity, (**b**) Swelling ratio, and (**c**) water uptake. Different amount of TiO_2_ nanoparticles in 5 h sPEEK/TiO_2_ composite membranes: (**d**) proton conductivity, (**e**) vanadium ion permeability, (**f**) permeability and selectivity. VRFB cell performance of 5 h sPEEK/ 5% TiO_2_ composite membranes: (**g**) charge-discharge cycle test, (**h**) efficiency, (**i**) normalized discharge capacity and (**j**) efficiencies at different current density. Reprinted with permission from ref. [141]. Copyright © 2017 Elsevier B.V. (License Number: 5270230374353).

In this connection, Ji et al. used TiO_2_ inorganic filler for preparing a uniform sPEEK/TiO_2_ composite membrane by optimizing DS and TiO_2_ with a solvent casting method using DMSO solvent [141]. Increasing the sulfonation time increases the IEC, which increases the membrane’s water uptake and swelling ratio. It has been shown in the order of 7 h sPEEK > 5-h sPEEK > 3 h sPEEK. Increased proton conductivity is also enhanced by a high water swelling ratio, but this decreases the membrane’s mechanical strength. Here, 5 h sPEEK exhibited a similar water uptake and swelling ratio as Nafion 117 membrane. The high loading of TiO_2_ nanoparticles incorporated into the membrane decreases water uptake, swelling ratio, IEC, and IC (Figure 4a–d). Excessive TiO_2_ acts as a blocking agent, decreasing the swelling ratio and free volume in the membrane. Thus, it prevents proton transport across the membrane. Although 5 h sPEEK/5% TiO_2_ loading exhibited an identical proton conductivity value of 17.6 mS cm^−1^ as Nafion 117. Additionally, the composite membrane showed better oxidative and chemical stability in VRFB. The TiO_2_ nanoparticle incorporation also reduces the vanadium ions permeation due to the barrier effect that creates a smaller water channel. It has been observed the 5 h sPEEK/5% TiO_2_ composite membrane showed 2.45 × 10^−7^ cm^2^ min^−1^ of vanadium permeation lower than Nafion 117 as represented in Figure 4e,f. Additionally, the good dispersion of TiO_2_ with sulfonic acid backbone attributed become the increased selectivity in the 5 h sPEEK/5% TiO_2_ composite membrane. The self-discharge of VRFB with the 5 h sPEEK/5% TiO_2_ composite membrane by charging to SOC of 50% has been evaluated. Initially, the OCV decreases slowly to about 1.2V and then drops sharply to 0.8V. Hence, the 5 h sPEEK/5% TiO_2_ composite membrane (107 h) exhibited three times higher self-discharge due to vanadium crossover than Nafion 117 (34 h). It has been observed that there was no decline in charge and discharge capacity with the increased cycles during VRFB single-cell operation with the composite membrane, as shown in Figure 4g–j. Furthermore, VRFB with the Nafion 117 with 180 µm thickness (0.27% per cycle) exhibited two times higher decay rates than the composite membrane with 118 µm thickness (0.11% per cycle). The composite membrane exhibited higher CE and EE than the Nafion membrane. In addition, the 5 h sPEEK/5% TiO_2_ composite membrane is intact after 100 cycles, which is evidence of higher stability [141].

To improve the mechanical strength and vanadium permeation resistivity, an idea of different kinds of inorganic nanoparticles (Al_2_O_3_, SiO_2,_ and TiO_2_) incorporated into the sPEEK membrane for composite membrane preparation (S/A, S/S, and S/T) by solution casting method at different concentrations (2.5%, 5%, 7.5% and 10%) has been developed [124]. Further, the introduction between the –SO_3_H group of sPEEK and the OH group of inorganic nanoparticles strengthens the compatibility of the composite membrane. Their dense and smooth surface identifies the uniform distribution of inorganic nanoparticles in the composite membrane without a pinhole. The composite membrane exhibited a more decreased WU value compared to sPEEK, but more than Nafion 117. In the same way, the composite membrane exhibited SR of S/A-10% (17.7%), S/S-10% (17.5%), and S/T-10% (19.3) lower than the sPEEK (26.5%) membrane due to the low content of sPEEK and inorganic nano-oxides interaction. These decreasing results are attributed to high mechanical strength for composite membranes with increased dimensional stability. Additionally, the composite membrane also experiences lower proton conductivity and IEC value due to the reduction of free sulfonic acid groups by higher loading of nano-oxides. The OH groups in nano-oxides occupied the free –SO_3_H groups of sPEEK by intermolecular H_2_ bonding. Consequently, the composite membranes exhibited low vanadium ion permeability due to lower WU and IEC. The nano-oxides in the composite membrane act as a barrier for proton transport and suppress the vanadium ion permeability. Due to the incorporation of Al_2_O_3_, SiO_2,_ and TiO_2_, the composite membranes showed enhanced breaking strength with decreasing elongation percentage that increases the membrane mechanical strength. Furthermore, the 5% loading of nano-oxides in sPEEK exhibited a proper balance between physicochemical and mechanical properties for VRFB application. The VRFB single cell performance with composite membranes has been evaluated at various current densities (40 to 200 mA cm^−2^). The composite membranes S/A-2.5%, S/A-5%, S/A-7.5% and S/A-10% exhibited an increasing CE than Nafion 117 due to their low vanadium ion crossover. As a result of decreased vanadium ion crossover and increased ionic selectivity, the S/A-5% (85.0%), S/S-5% (84.8%), S/T-5% (83.5%) exhibited higher EE than Nafion 117 (79.5%) at 80 mA cm^−2^. The composite membrane exhibited good self-discharging time due to low VO^2+^ permeability, SR, and high mechanical strength. Because of Al_2_O_3_ (13 nm size), proper dispersion in sPEEK membrane, S/A-5% membrane possesses a longer self-discharge time (170 h) than the Nafion 117 (46 h) and sPEEK (80 h). The composite membrane S/A-5% and S/T-5% remain at 50% capacity after 200 cycles, whereas the Nafion 117 membrane loses more than 75% capacity indicating the composite membrane has higher stability [124].

**Figure 5:**
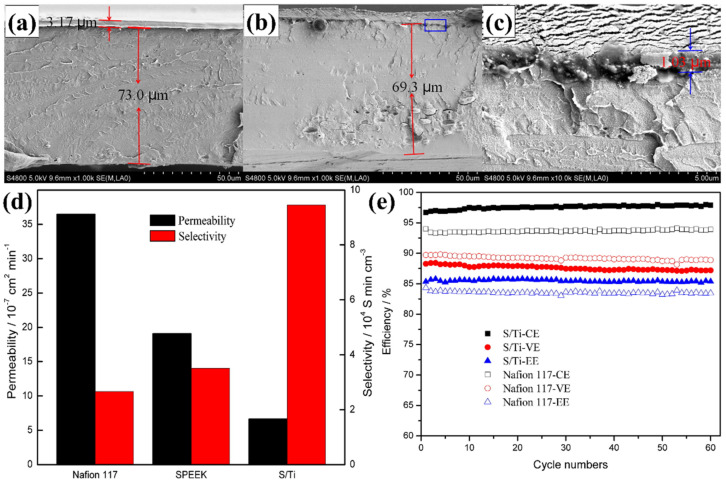
SEM cross-sectional image of (**a**) before and (**b**,**c**) after cell test of sPEEK/TiO_2_ double-deck membrane. (**d**) Vanadium ion permeability and selectivity (**e**) cycle performances (efficiency) of different membranes. Reprinted with permission from ref. [140]. Copyright © 2016 Elsevier B.V. (License Number: 5270241140135).

In another study, a novel sPEEK/TiO_2_ double-deck (S/T) membrane was prepared for VRFB using the solvent casting method [140]. In this study, a TiO_2_ inorganic particle was used to improve the antioxidant ability and overall membrane stability of the non-fluorinated sPEEK membrane. From Figure 5a–c, SEM analysis indicated the membrane double-deck structure without cracks that indicated the successful deposition of TiO_2_ layer on sPEEK membrane holes. In the S/T membrane, the TiO_2_ layer deposition on the sPEEK membrane reduces the H_2_O diffusion channel and thus decreases H_2_O absorption. Therefore, the S/T membrane exhibited a lower H_2_O uptake and swelling ratio. Furthermore, the S/T membrane displayed decreased proton conductivity due to the proton-nonconductive TiO_2_ layer. The vanadium permeability of the S/T membrane (6.66 × 10^−7^ cm^2^ min^−1^) is lower than Nafion 117 (36.5 × 10^−7^ cm^2^ min^−1^) and sPEEK (19.1 × 10^−7^ cm^2^ min^−1^). This was due to the distribution of the TiO_2_ layer on the sPEEK membrane that reduces the ion transport channel with increased dead-end pockets that prevent vanadium ion crossover. Comparatively, the S/T membrane exhibited higher selectivity of 9.46 × 10^4^ S min cm^−1^ than the Nafion 117 membrane due to lower permeability (Figure 5d). The VRFB cell with an S/T membrane exhibited higher CE (97.0%) and EE (85.8%) higher than the Nafion 117 membrane (CE, 93.3% and EE, 83.7%) as shown in Figure 5e. In addition to this, the composite membrane showed a lower decay rate than Nafion 117, with outstanding VRFB cycle performances [140].

Lou et al. prepared an sPEEK/TiO_2_ composite membrane to overcome the stability-related issues and efficient VRFB performances by solvent casting method [142]. TiO_2_ has been selected in this direction due to its cost-effectiveness, feasibility, and super hydrophilicity. Thus it improved the proton conductivity, ionic selectivity, and chemical stability. The uniform and successful dispersion of TiO_2_ nanoparticles in the sPEEK matrix has been effectively achieved. The size and uniformity of TiO_2_ filler into sPEEK altered the water channel size and suppressed the vanadium transport by repulsing the larger stoke radius vanadium ion. Hence, the sPEEK/TiO_2_-5% (0.18 × 10^−7^ cm^2^ min^−1^) membrane exhibited lower vanadium ion permeability than the Nafion 212 (1.87 × 10^−7^ cm^2^ min^−1^) and sPEEK (0.35 × 10^−7^ cm^2^ min^−1^). On the other hand, sPEEK/TiO_2_-5% showed decreased proton conductivity (18.3 mS cm^−1^) compared to the sPEEK membrane (25.28 mS cm^−1^) due to TiO_2_ filling into the sPEEK composite membrane. The electrochemical performance of the VRFB unit cell with composite membrane showed a considerable charge-discharge performance at 50 mA cm^−2^ current density. As a result, the composite membrane exhibited a higher charge voltage than Nafion 212 and oppositely exhibited a discharge process. This was due to the higher thickness of the sPEEK/TiO_2_-5% membrane, in which the proton transport pathway was elongated. Furthermore, the composite membrane showed a high discharge time of 87.5 h higher than the Nafion 212 membrane, which showed only 15.7 h. In addition to this, the composite membrane showed a higher CE of 99.3% due to decreased vanadium permeability. An increase in current density means the VE and EE of both composite membrane and Nafion 212 start decreasing. The reason behind this implied the increased ohmic resistance and potential due to increased current density. During long cycle operation at 120 mA cm^−2^, the composite membrane exhibited outstanding CE (over 99.3%), EE (84.8%) with a discharge capacity of 95.4% at 100th cycle and 86.8% at the 200th cycle. Therefore, the composite membrane exhibited the highest stability with enhanced mechanical strength and ionic selectivity due to inorganic TiO_2_ nanoparticles [142].

Further altering the sPEEK/TiO_2_ composite has enhanced performances in VRFB membranes. The concepts of incorporation of TiO_2_ with graphene oxide (GO) and TiO_2_ sulfonation have shown considerable attention in preparing the hybrid membranes. The nanosheet GO and titanium dioxide nanoparticle (TiO_2_) inorganic nanofillers are introduced into the sPEEK matrix to develop the sPEEK/GO/TiO_2_ hybrid membrane by the solvent casting method [143]. The blocking effect caused by GO is responsible for vanadium permeation, whereas the GO nanosheets modification performed by TiO_2_ aids proton transportation. Additionally, the GO inoxidizability influences the membrane dimensional stability by reinforcing the membrane. In order to increase the hybrid membrane thickness, the various amounts of nanoparticles also increased (0.5 GO, 0.5T, 1.0T, 2.0T). The blocking effect of S/0.5GO/0.5T exhibited decreased vanadium permeability (~0.23 × 10^−7^ cm^−2^ min^−1^) which is 1/7th of Nafion (~1.6 × 10^−6^ cm^−2^ min^−1^) that is due to the GO nanosheets and TiO_2_, which acts as a blocker for vanadium ions transport in the membrane. Further, increased TiO_2_ particle loading created increased vanadium ions cross over because a large number of TiO_2_ nanoparticles aggregated and developed big pores in the hybrid membrane. The prolonged iron transport channel developed by membrane thickness directly influences membrane resistance. The increase in additive loading decreases the swelling ratio of the hybrid membrane, which increases membrane stability. The ultrasonic and magnetic stirring method introduced TiO_2_ into the GO sheet, developing a small ions transport channel that transports only ions with a small stroke radius and hence decreases vanadium ion transport. The hybrid membrane S/0.5GO/0.5T exhibited the highest CE of ~99.2% than Nafion (96.2%) due to the blocking effect of GO and TiO_2_ nanoparticles. Additionally, the S/0.5GO/0.5T hybrid membrane showed ~97.20% of discharge capacity even after 100 cycles with outstanding cycle performances [143].

**Figure 6:**
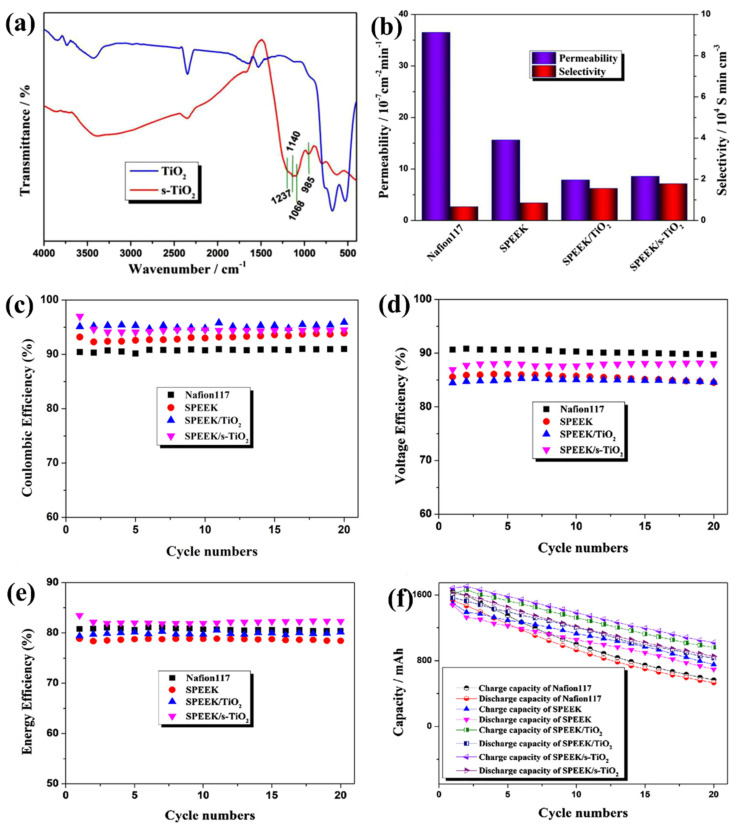
(**a**) FT-IR spectra of TiO_2_ before and after sulphonation. (**b**) Vanadium ion permeability and selectivity, (**c**) coulombic efficiency, (**d**) voltage efficiency, (**e**) energy efficiency, and (**f**) cycle stability of membranes (Nafion117, sPEEK, sPEEK-TiO_2_, and sPEEK-s-TiO_2_). Reprinted with permission from ref. [126]. Copyright © 2019 Wiley Periodicals, Inc. (License Number: 5270521350593).

The development of wide molecular and ion channels by the high degree of sulfonation in sPEEK membrane resulted in high vanadium permeability and low mechanical strength. To overcome this fact, the inorganic fillers are introduced into the sPEEK membrane. In most cases, the inorganic fillers in the sPEEK membrane reduce the membrane proton conductivity. Therefore, functional groups, namely –SO_3_H, –COOH, and –NH_2_ are added as inorganic fillers by oxidation, sulfonation, and acidification reaction. In this way, the –SO_3_H group has been introduced into TiO_2_ nanoparticles by the sulfonation process for the preparation of the sPEEK/sulfonated TiO_2_ composite membrane for VRFB application [126]. As shown in Figure 6a, the sulfonation functional group in TiO_2_ nanoparticles has been successfully confirmed by IR analysis. The –SO_3_H group not only optimizes the ionic channels but also increases the composite membrane proton conductivity. The enhancement in composite membrane thermal stability was due to the intermolecular hydrogen bond formation by –SO_3_H addition to TiO_2_ in sPEEK/s-TiO_2_ membrane. Furthermore, the –SO_3_H group in TiO_2_ opens the more ion transport channels, resulting in higher proton conductivity. This results in a low vanadium permeability, but not as low as the Nafion 117, sPEEK, and sPEEK/TiO_2_ membrane. The sPEEK/sTiO_2_ composite membrane (7.13 × 10^4^ S min cm^−3^) exhibited two times higher selectivity than the sPEEK membrane (3.40 × 10^4^ S min cm^−3^) and three times than Nafion 117 (2.66 × 10^4^ S min cm^−3^) as shown in Figure 6b. The VRFB with the sPEEK/sTiO_2_ composite membrane exhibited the best charge-discharge performance (Figure 6c–f). Moreover, the sPEEK/sTiO_2_ provided a high EE of 82.3% and high discharge capacity because of the high selectivity of the membrane. The efficient unit cell performance is mainly attained because of the presence of sulfonated TiO_2_ in the membrane matrix, where sulfonated TiO_2_ acts as a barrier for vanadium ion permeability and further improves the proton transportation with the existence of –SO_3_H in TiO_2_ [126].

**Figure 7:**
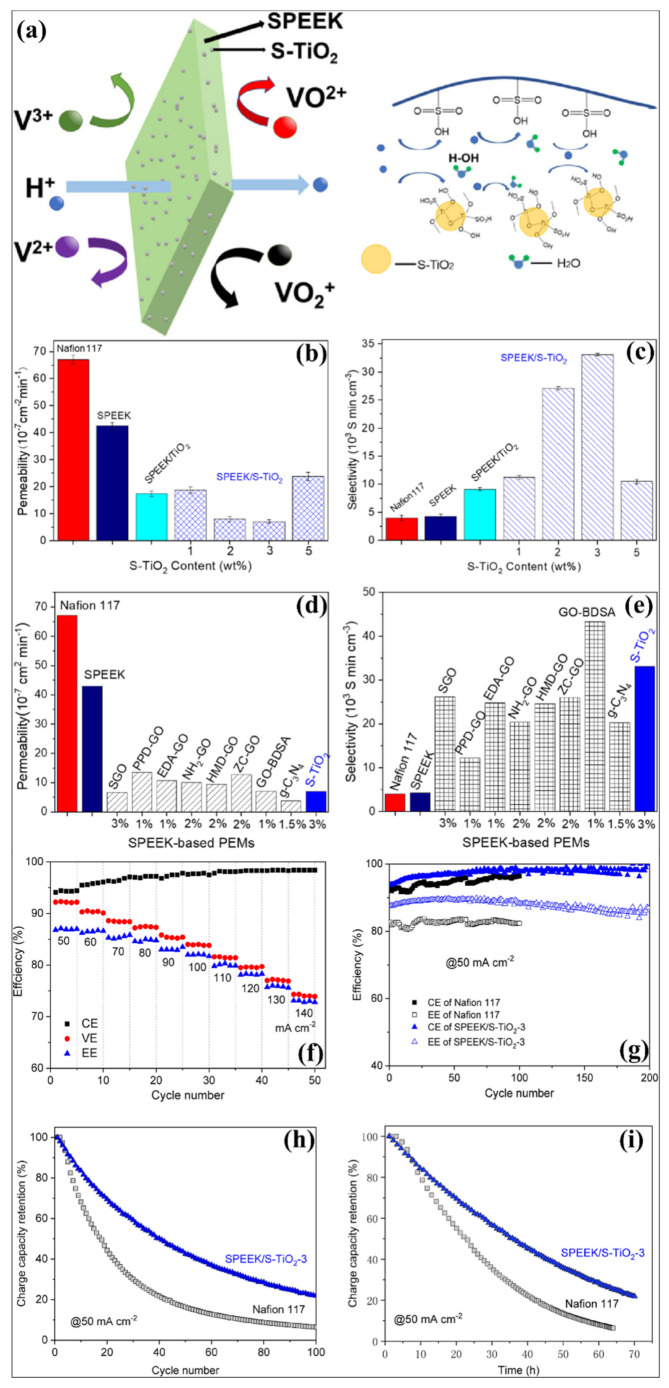
(**a**) Schematic representation of proton transport mechanism in the sPEEK-sTiO_2_ membrane. (**b**) Vanadium ion permeability and (**c**) selectivity of sPEEK membrane with different loading of sTiO_2_. (**d**) Vanadium ion permeability and (**e**) selectivity of sPEEK-sTiO_2_ membrane compared with other reported results. (**f**–**i**) VRFB unit cell performances of sPEEK-sTiO_2_ hybrid membrane. Reprinted with permission from ref. [125]. Copyright © 2021 Elsevier Ltd. (License Number: 5270530067581).

Adding acidic groups to nanoparticle surfaces reduces the swelling properties and vanadium iron crossover with improved ionic conductivity in the membrane as shown in Figure 7a. The sulfonation on the particle surface -OH groups resulted in the -SO_3_H functionalization. In the same way, TiO_2_ was sulfated to sTiO_2_ with enhanced acidic properties and has been incorporated at a low loading ratio into the sPEEK matrix by the solution casting method, used for obtaining a hybrid membrane with enhanced selectivity ionic conductivity and mechanical stability [125]. The sulfonation of TiO_2_ was effectively introduced in the sPEEK. The increased hydrophilic properties of sPEEK in the hybrid membrane due to the sTiO_2_ incorporation enhance the WU due to a higher concentration of H^+^. Moreover, the SR of the hybrid membrane due to sTiO_2_ fillers on the sPEEK matrix acts as a barrier. The enhanced amount of SO_3_H groups in the hybrid membrane due to sTiO_2_ in the hybrid membrane possesses an increased conductivity (20.9–25.1 ± 0.2 mS cm^−1^) compared to sPEEK (16.4 ± 0.3 mS cm^−1^), sPEEK/TiO_2_ (15.9 ± 0.2 mS cm^−1^) and IEC. The sPEEK/sTiO_2_ −3(3% sTiO_2_) exhibits a lower vanadium permeation (7.0 × 10^−7^ cm^−2^ min^−1^) than sPEEK/TiO_2_ (17.4 × 10^−7^ cm^−2^ m^−1^) as shown in Figure 7b–e. This was due to the restraining vanadium ion movement and also the stabilization of proton transport channels by –SO_3_ H groups of sTiO_2_. The improvement in IEC and proton conductivity has been attributed to the hydrophilicity developed by −OH and −SO_3_H groups (scheme). The decreased vanadium ion permeability of the sPEEK/sTiO_2_-3 hybrid membrane exhibited an increased self-discharge time of 55 h, which was higher than Nafion 117 (33 h), sPEEK (37 h), sPEEK/TiO_2_ (41 h). The VRFB unit cell with sPEEK/sTiO_2_-3 hybrid membrane showed increased CE and EE as shown in Figure 7f,g. The membrane mechanical stability and chemical stability were enhanced by an intramolecular interaction between OH and -SO_3_H groups in the hybrid membrane [125]. The TiO_2_-based inorganic nanofillers have effectively altered the physicochemical properties of membranes and VRFB unit cell performances, as represented in Table 1 and Table 2. The introduction of TiO_2_ in the polymer matrix effectively controls the higher amount of water uptake by the membrane and the swelling ratio of the membrane. More interestingly, the vanadium ion permeability is significantly lowered to the Nafion, SPI and sPEEK membranes after introducing of TiO_2_ in the membrane. Thus, the TiO_2_ nanoparticles in the membrane acted as a barrier for the easier movement of vanadium ions through ionic clusters, which is the primary reason for obtaining the lower permeability. With the further modification of TiO_2_ as a sulfonation and other organic compounds, the proton conductivity and stability have been further tuned. Based on the reasonable proton conductivity and lower vanadium ion crossover, most of the TiO_2_-containing membranes achieved higher selectivity than the bare polymer membrane. Thus, the TiO_2_-based hybrid membrane revealed excellent VRFB performances during the unit cell operation. Apart from the membrane applications, TiO_2_ has been widely considered an efficient electrode material for various applications such as photoelectrochemical devices, batteries, and fuel cells [152,153,154]. The different concepts and structural properties of TiO_2_ nanostructures developed for photoelectrochemical devices [152,155,156,157] can be further altered and considered as a new kind of TiO_2_-based additive for VRFB membranes. This comprehensive review will be more resourceful for the polymer and membranes scientific community to choose the further development in the TiO_2_-containing polymer membranes for VRFB and other energy conversion and storage systems.

## 7. Conclusions

VRFB has gained great attraction in extensive scale energy storage systems due to its long cycle life, easy maintenance, and high efficiency. In VRFB, ion exchange membrane (IEM) plays a key role in battery performance. An excellent IEM should possess high proton conductivity, good chemical stability, low vanadium permeability, low cost, high mechanical strength, etc. DuPont Nafion perfluorinated sulfonic acid polymer membrane has been commonly used in VRFB due to its increased proton conductivity and chemical stability. Nafion membranes have limitations in VRFB due to their high cost and high vanadium permeability. To overcome these issues, incorporating inorganic nanoparticles into Nafion has been performed to decrease vanadium permeability. This comprehensive study reviewed TiO_2_ as an inorganic filler for VRFB membranes. It has been confirmed that TiO_2_ is one of the best inorganic fillers due to its low price, high stability, and easy preparation method. The TiO_2_ occupies the Nafion matrix, thus decreasing the swelling ratio by reducing the size of the water transport channel. Thus, it decreases the vanadium ion cross-over. Modification such as organic silica modified TiO_2_ has also been introduced into Nafion to increase the chemical stability in a high acidic electrolyte environment with decreased vanadium ion permeability. Apart from this, different non-perfluorinated polymer membranes (sPEEK, sPI, etc.), have also been developed as IEM as an alternative to the Nafion membrane. Low chemical stability and high vanadium permeation were identified as the major drawback of this type of hydrocarbon membrane (such as sPEEK). The DS in the sulfonated membranes increased the proton conductivity but also increased vanadium ion permeability. Therefore, such membranes are modified by incorporating inorganic nanofillers and modified nanofillers to enhance membrane performances. However, nanofiller introduction decreases the proton conductivity by occupying the SO_3_H groups. Therefore, functional group modification (SO_3_H, COOH, NH_2_) on nanofiller has been accomplished to enhance membrane proton conductivity. The sulfonation of TiO_2_ alters the ion transport channels and increases proton conductivity. In addition to this, the nanoparticle incorporation increases mechanical stability, suppresses the vanadium ion permeability, enhances selectivity, and improves the overall VRFB unit cell performances. Hence, this review encompasses the overall developments in TiO_2_-based hybrid composite membranes for VRFB applications.

## Figures and Tables

**Table 1:** Physicochemical properties of different polymers with TiO_2_ nanofillers as a hybrid membrane for VRFB.

Hybrid Polymer Composite	Thickness (µm)	WU (%)	IEC (mmol/g)	Area Resistance (Ω cm^2^)	IC (mS/cm)	Permeability (cm^2^/min)	Selectivity (S min cm^−3^)	Ref.
Nafion	215	26	0.97	1.14	-	36.9 × 10^−7^	-	[138]
Nafion/Si/Ti	225	22.5	0.95	1.26	-	4.3 × 10^−7^	-	
Nafion	89	21.15	0.88	1.04	-	2.26 × 10^−5^	-	[123]
Nafion/TiO_2_	90	19.13	0.85	1.05	-	6.72 × 10^−6^	-	
Nafion212	50	~13.9	-	0.320	15.5	~1.75 × 10^−7^	8.32 × 10^4^	[68]
rN212	36.1 ± 0.3	~6.8	-	0.225	16	~1.8 × 10^−7^	-	
rN212/T0.5	37.0 ± 0.3	~5.5	-	0.266	13.8	~1.55 × 10^−7^	-	
rN212/T1.0	38.1 ± 0.3	~4.5	-	0.294	13	~1.4 × 10^−7^	-	
rN212/T1.5	39.1 ± 0.3	~4	-	0.317	12.7	~1.15 × 10^−7^	10.68 × 10^4^	
rN212/T2.0	40.0 ± 0.2	~3.4	-	0.414	9.8	~ 1.3 × 10^−7^	-	
rN212/T2.5	41.2 ± 0.3	~2.8	-	0.536	7.6	~1.35 × 10^−7^	-	
Nafion 117	175	19.42	1.132	-		17.1 × 10^−7^	0.34 × 10^5^	[139]
SPI	55	38.46	1.404	-		1.9 × 10^−7^	1.30 × 10^5^	
sPI/TiO_2_	49	32.94	1.243	-		2.02 × 10^−7^	1.54 × 10^5^	
Nafion 117	220	28.5	0.91	-	97	36.5 × 10^−7^	2.66 × 10^4^	[140]
sPEEK	107	56.6	1.81	-	67	19.1 × 10^−7^	3.51 × 10^4^	
S/Ti	90	40.4	1.58	-	63	6.66 × 10^−7^	9.46 × 10^4^	
Nafion 117	215	33	0.86		90	32 × 10^−7^	26.5 × 10^3^	[124]
sPEEK	65	62.6	2.24		23	9.7 × 10^−7^	25. 5× 10^3^	
S/T-2.5%	64	55.5	2.12		20	-		
S/T-5%	68	60.6	2.06		18	4.4 × 10^−7^	40.9 × 10^3^	
S/T-7.5%	59	59.1	1.96		13	-		
S/T-10%	69	54.8	1.75		8	-		
Nafion 117	180	26.93	-	-	17.6			[141]
5 h sPEEK	172	24.23	-	-	-	3.24 × 10^−6^	5.42 × 10^3^	
5 h sPEEK-3% TiO_2_	178	-	-		21.1	-	-	
5 h sPEEK-5% TiO_2_	118	-	-	-	17.8	2.45 × 10^−7^	72.55 × 10^3^	
5 h sPEEK-7% TiO_2_	180	-	-	-	14.3	-	-	
Nafion 212	50	-	-	0.252	19.8	1.87 × 10^−7^	10.59 × 10^3^	[142]
sPEEK	72	-	-	0.285	25.28	0.35 × 10^−7^	72.22 × 10^3^	
sPEEK—TiO_2_	82	-	-	0.448	18.3	0.18 × 10^−7^	101.67 × 10^3^	
Nafion 117	220	28.5	0.91	-	97	36.5 × 10^−7^	2.66 × 10^4^	[126]
sPEEK	99	28.8	1.49	-	53	15.6 × 10^−7^	3.4 × 10^4^	
sPEEK/TiO_2_	106	26.6	1.42	-	49	7.86 × 10^−7^	6.23 × 10^4^	
sPEEK/s-TiO_2_	93	30.1	1.59	-	61	8.55 × 10^−7^	7.13 × 10^4^	
Nafion 117	-	21.1 ± 0.3	0.88 ± 0.1	-	26.8 ± 0.3	67.2 × 10^−7^	4 × 10^3^	[125]
sPEEK	-	37.0 ± 0.3	1.88 ± 0.1	-	16.4 ± 0.3	43.0 × 10^−7^	4.3 × 10^3^	
sPEEK/TiO_2_	-	38.4 ± 0.1	1.77 ± 0.1	-	15.9 ± 0.2	17.4 × 10^−7^	10.8 × 10^3^	
sPEEK/S-TiO_2_-1	-	42.4 ± 0.2	1.89 ± 0.1	-	20.9 ± 0.2	-	-	
sPEEK/S-TiO_2_-2	-	43.2 ± 0.2	1.92 ± 0.1	-	21.7 ± 0.2	-	-	
sPEEK/S-TiO_2_-3	-	44.5 ± 0.2	1.95 ± 0.1	-	23.2 ± 0.2	7.0 × 10^−7^	33.1 × 10^3^	
sPEEK/S-TiO_2_-5	-	47.8 ± 0.1	2.00 ± 0.1	-	25.1 ± 0.2	-	-	

**Table 2:** VRFB unit cell performances (efficiencies) of different kinds TiO_2_ based nanofillers incorporated hybrid membranes.

Materials	Current Density (mA/cm^2^)	CE (%)	VE (%)	EE (%)	Ref.
Nafion	30	90.8	84.8	77	[138]
Nafion/Si/Ti		94.8	82.2	77.9	
Nafion	60	86.3	80.6	69.6	[123]
Nafion/TiO_2_		88.8	80.5	71.5	
Nafion N212	120	94.5	-	79.2	[68]
rN212/T1.5		98.3	-	84.4	
Nafion 117	20	80.6	71	88.1	[139]
SPI/TiO_2_		93.8	83	88.5	
Nafion 117	60	93.3	-	83.7	[140]
S/Ti		97	-	85.8	
Nafion 117	50	96.2	85	81.8	[141]
5 h sPEEK-5% TiO_2_		98.3	84.4	82.9	
Nafion 117	50	90.03	90.07	~80.08	[126]
sPEEK		~93	~85.2	~79	
sPEEK/TiO_2_		95.3	84.9	~79.5	
sPEEK/s-TiO_2_		~94.5	~87	82.3	
Nafion 117	100	97.1	-	74.1	[125]
sPEEK		98	-	72.3	
sPEEK/TiO_2_		98.1	-	71.3	
sPEEK/S-TiO_2_-3		98.8	-	80.2	

## Data Availability

Not applicable.
